# Clinical Application of Physical Therapy in Neurorehabilitation

**DOI:** 10.3390/jcm12082752

**Published:** 2023-04-07

**Authors:** Sofia Straudi, Roberto Cano-de-la-Cuerda

**Affiliations:** 1 Department of Neuroscience and Rehabilitation, Ferrara University, 44121 Ferrara, Italy; 2 Department of Physical Therapy, Occupational Therapy, Physical Medicine and Rehabilitation, Rey Juan Carlos University, 28922 Madrid, Spain

The knowledge from basic neuroscience studies on mechanisms of motor recovery and the development of theoretical models of learning and recovery has favoured the development and implementation of neurophysiologically sounded rehabilitative interventions. Even if no clear conclusions have been made, the influence of relevant ingredients on functional and global outcomes has been proposed, such as the treatment dose, intensity, repetition, specificity of the functional task proposed, motivation, engagement, and feedback used. Highly repetitive, task-specific training is necessary to induce cortical reorganization and promote neural plasticity [1]. However, even if repetitions matter, they are not enough to be effective. Another critical element of motor recovery is the motivation and cognitive engagement of a patient receiving rehabilitation [2]. Finally, the learning process can also depend on the feedback provided (implicit or explicit) and its frequency. For this reason, a theoretical decalogue in rehabilitation is available to induce neural plasticity with interventions [3].

Since the 1990s, technology has been increasingly introduced in clinical settings as a tool to augment the intensity of neurorehabilitation and foster motor recovery. Moreover, technologies might permit manipulating recovery’s key ingredients, delivering an assisted, massed practice in an enriched environment. A clear example of translating basic neuroscience principles into clinical practice is the mirror neuron system-based rehabilitation techniques, such as action observation therapy (AOT), motor imagery, and mirror therapy. Activating specific brain areas during action observation has been seen as a potentially powerful rehabilitation target to foster motor recovery [4].

Robot-assisted therapy (RAT) is one of the most widely used technologies in clinical settings for restoring gait and arm function. It can increase the amount of therapy, providing an enriched therapeutic scenario using gaming or virtual reality systems. Robotic devices can be classified as end-effectors or exoskeletons. Other classifications, mainly focusing on gait rehabilitation, can attempt their portable or not-portable characteristics. The end-effectors control only the distal part of the limb, whereas the exoskeletons with actuators can handle multiple joints and train an increased complexity of movements [5]. So far, international guidelines for stroke patients have included RAT for promoting recovery. For upper limb robotics, activities of daily living, arm function, and arm muscle strength might improve in stroke patients [6].

Concerning gait rehabilitation, patients who received electromechanical-assisted gait training in combination with physiotherapy after stroke were more likely to walk independently than patients who received gait training without these devices [7].

However, the applicability of these indications is still minimal, with little information on the proper training protocol, dosage, and profile of the responders [8,9]. Moreover, a longer step has to be taken to develop a new generation of devices with a clear neurophysiological and biomechanical rationale of use [10], estimating the costs (or savings) due to electromechanically assisted training and for how long the benefits last.

Among technology-assisted interventions, the gamification of therapy is one of the most relevant strategies conceptualized in the last decade to promote the enjoyment and reward of neurological patients. So far, gaming has been combined with robotics or utilised alone with exergaming platforms or, more recently, creating specific virtual reality (VR) scenarios [11].

Relevant elements related to VR are *immersion* and *interaction,* which refers to the level of presence, plausibility, and embodiment achieved during training. Specifically, *immersion* is the ability to elicit in the patient a perception of “being real” and to be part of the training received. High variability in the degree of immersion is present in the available systems, ranging from immersive VR with a head-mounted display to semi-immersive with a single-screen projection and non-immersive using a pad or computer desktop. The interaction between the patient and the VR scenario, as well as the level of physical activity generated, depends on the hardware and software of the VR system that can be very simple (i.e., joystick) or more complex (i.e., sensors, haptic feedback devices). [12]. VR also might add key elements for motor learning in stroke patients (but not exclusively) as specific practice, explicit or implicit feedback, increasing difficulty, variable practice and forced use [13]. Scientific evidence demonstrates that when virtual reality is used in addition to usual care or rehabilitation in stroke patients to increase the amount of time the person spends in therapy, there are improvements in function [14]. Above all, semi-immersive and immersive VR systems seem promising for tailoring rehabilitation to the patient’s specific needs and outcomes, even at home [15]. Given the limited resources for healthcare and the window of opportunities for recovery, developing solutions available remotely (telerehabilitation) is timely and meaningful. Indeed, more recently, technology has become even more digital (e.g., mobile applications or wearable sensors), opening the door to a new treatment scenario encompassing the patient’s home and the community for a more sustainable rehabilitation model. Regarding mobile applications, several apps have been done with the aim of promoting real-time communication, remote assessment, and training of specific domains (i.e., balance, healthy lifestyle). However, this scientific field is still in its infancy with limited validity and applicability in clinical practice [16].

Contemporary neurorehabilitation would consider all the potential contributors to the recovery process, including the long-term management of motor disabilities and the promotion of self-management and empowerment of patients and caregivers.
